# Monitoring the Size and Lateral Dynamics of ErbB1 Enriched Membrane Domains through Live Cell Plasmon Coupling Microscopy

**DOI:** 10.1371/journal.pone.0034175

**Published:** 2012-03-28

**Authors:** Guoxin Rong, Björn M. Reinhard

**Affiliations:** Department of Chemistry and The Photonics Center, Boston University, Boston, Massachusetts, United States of America; University of Patras, Greece

## Abstract

To illuminate the role of the spatial organization of the epidermal growth factor receptor (ErbB1) in signal transduction quantitative information about the receptor topography on the cell surface, ideally on living cells and in real time, are required. We demonstrate that plasmon coupling microscopy (PCM) enables to detect, size, and track individual membrane domains enriched in ErbB1 with high temporal resolution. We used a dendrimer enhanced labeling strategy to label ErbB1 receptors on epidermoid carcinoma cells (A431) with 60 nm Au nanoparticle (NP) immunolabels under physiological conditions at 37°C. The statistical analysis of the spatial NP distribution on the cell surface in the scanning electron microscope (SEM) confirmed a clustering of the NP labels consistent with a heterogeneous distribution of ErbB1 in the plasma membrane. Spectral shifts in the scattering response of clustered NPs facilitated the detection and sizing of individual NP clusters on living cells in solution in an optical microscope. We tracked the lateral diffusion of individual clusters at a frame rate of 200 frames/s while simultaneously monitoring the configurational dynamics of the clusters. Structural information about the NP clusters in their membrane confinements were obtained through analysis of the electromagnetic coupling of the co-confined NP labels through polarization resolved PCM. Our studies show that the ErbB1 receptor is enriched in membrane domains with typical diameters in the range between 60–250 nm. These membrane domains exhibit a slow lateral diffusion with a diffusion coefficient of 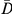 = |0.0054±0.0064| µm^2^/s, which is almost an order of magnitude slower than the mean diffusion coefficient of individual NP tagged ErbB1 receptors under identical conditions.

## Introduction

The signaling activity of members of the epidermal growth factor receptor family, which comprises the receptors ErbB1-4 [1], [2], does not only depend on the association of the receptors into discrete molecular species, such as dimers [3], [4] and potentially higher order oligomers [5]–[7], but also on the self-organization of the receptors on longer (i.e. tens to hundreds of nanometer) spatial length scales [8]. The enrichment of the receptors in “microdomains” [9]–11 or “nanoclusters” [12], [13] is anticipated to influence the dynamic equilibrium between the receptors and receptor assemblies [14]–[17]. Although the exact relationship between the topography of the ErbB receptor enrichment and the signaling activity is not accurately understood, it is clear that the geometric size, shape, and number of receptors of individual signaling domains determine the local receptor density. The latter will influence the receptor collision rate and could, therefore, have direct implications for the signaling activity, for instance, by shifting the local receptor association levels.

Different mechanisms can contribute to a heterogeneous distribution of ErbB receptors on the cell surface. It is conceivable that the transmembrane receptors become trapped in membrane compartments formed by actin [18], [19] or other non-actin (e.g. spectrin) [20]–[24] components of the cortical cytoskeleton. It has also been reported that the ErbB family members are enriched in spontaneously formed membrane compartments (“lipid rafts”) [25]–[27] that are the result of a dynamic self-organization of the membrane lipids. Finally, direct protein-protein interaction could stabilize extended ErbB aggregates formed in areas of high local ErbB concentration [20], [28]–[30]. It is possible that all of these effects contribute to the structuring of the spatial ErbB distribution, albeit on different length scales, and – unless otherwise noted - we refer in this manuscript to local enrichments in the ErbB concentration, independent of the exact formation mechanism, as “domains”.

The organization of ErbB receptor into signaling domains and transient fluctuations as well as systematic changes in the domain size and structure, for instance, in response to ligand addition, are very challenging to include in a rational analysis of signaling activity. The latter is primarily due to experimental difficulties associated with quantifying the structure, size, and spatial distribution of ErbB domains in living cells. The method of choice for characterizing the spatial distribution of individual components in living cells is light microscopy whose resolution limit (*d*) is defined by the wave nature of light. In conventional microscopy the resolution limit is given by 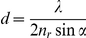, where λ is the wavelength of the light, n_r_ is the index of refraction of the ambient medium, and α is the angle of incidence. In the visible range of the electromagnetic spectrum *d* corresponds to approximately 250 nm, at best. Unfortunately, this is insufficient to probe the clustering of ErbB receptors on the nanometer to tens of nanometer length scale.

Specialized techniques enable to probe separations below *d*, and these techniques are very valuable tools for characterizing the self-organization of ErbBs. Fluorescence resonance energy transfer (FRET), for instance, utilizes non-radiative energy transfer between donor and acceptor dyes for optical distance measurements below the diffraction limit [13], [31]. The Förster critical distance lies, however, in the range between 2–6 nm for most organic dyes which limits the accessible distance range via FRET to very short separations and makes the analysis of the long-range organization of ErbBs difficult [32]. Superresolution fluorescence “nanoscopies” [33]–[35] can bridge the gap between the spatial FRET barrier and the diffraction limit, but both FRET and fluorescence nanoscopies suffer from the limited photostability and low signal intensities of organic dyes which limits the maximum observation time and localization precision at high temporal resolution. The characterization of the structural dynamics of laterally diffusing ErbB signaling domains remains, consequently, challenging, and important questions regarding the time-dependent structural organization of ErbB receptors remain to be addressed.

We have recently developed an alternative, non fluorescence based, approach for monitoring sub-diffraction limit separations in the optical microscope, called plasmon coupling microscopy (PCM) [36]–[38]. This method utilizes the distance dependent near-field coupling between noble metal nanoparticle (NP) labels to resolve close contacts on the length scale of approximately one NP diameter. PCM detects near-field interactions between discrete NPs in the far-field either as a spectral shift in the localized surface plasmon (LSP) resonance (LSPR) [39]–[41] or through changes in the polarization of the scattered light [36], [38]. Plasmon coupling based imaging modalities have been successfully applied by us [37], [42], [43] and others [44]–[46] to characterize the spatial distribution of NP immunolabels on cellular surfaces.

The use of NPs as labels in biological imaging benefits from the strong elastic scattering response [47], [48] that accompanies a resonant excitation of LSPs in metal NPs with diameters >20 nm. Since the NP label signal is based on scattering, NPs don't blink or bleach, which makes them attractive labels for high speed optical tracking with high localization precision [49]–[52]. The tracking of individual NP labeled receptors or lipids with high temporal resolution has already provided detailed insight into the structure of the plasma membrane in the past [19], [53]–[55]. In this work we augment the beneficial attributes of conventional NP tracking with the ability to resolve sub-diffraction limit contacts between NP immunolabels in PCM not only to detect and track membrane domains containing multiple NP labeled ErbB1 receptors but also to look “inside” the domains. The average separation of multiple NPs co-confined in one membrane domain depends on the size and shape of the confinement, and we show that the ability to monitor the configurational dynamics of the NP clusters within the membrane domains provides detailed structural information as function of time and location on the cell surface. To avoid any perturbation of the intricate cell membrane through chemical fixation, we performed our optical PCM studies on native membranes of living cells.

## Results and Discussion

### Optimization of NP Binding Affinity on Living Cells

Our rational for using plasmon coupling to localize plasma membrane domains enriched in ErbB1 receptors on epidermoid carcinoma cells (A431) is that areas of high local receptor concentration exhibit a higher binding affinity for immunolabels than adjacent areas with lower receptor concentration 11,12. Consequently, ErbB1 enriched membrane domains are expected to induce a local clustering of NPs, which is amenable to detection through PCM, provided that the NPs approach each other to distances below approximately one particle diameter. This experimental strategy requires the labeling of the receptors with Au NP immunolabels as a first step, which is not without complications. The glycocalyx coat [56], [57] on mammalian cells and the decrease in the structural flexibility that accompanies a tethering of binding chemistries (antibodies etc.) to NPs both interfere with the efficiency of the labeling. This is especially true for live cell studies, in which extensive washing and incubation times are not permissible.

**Figure 1:**
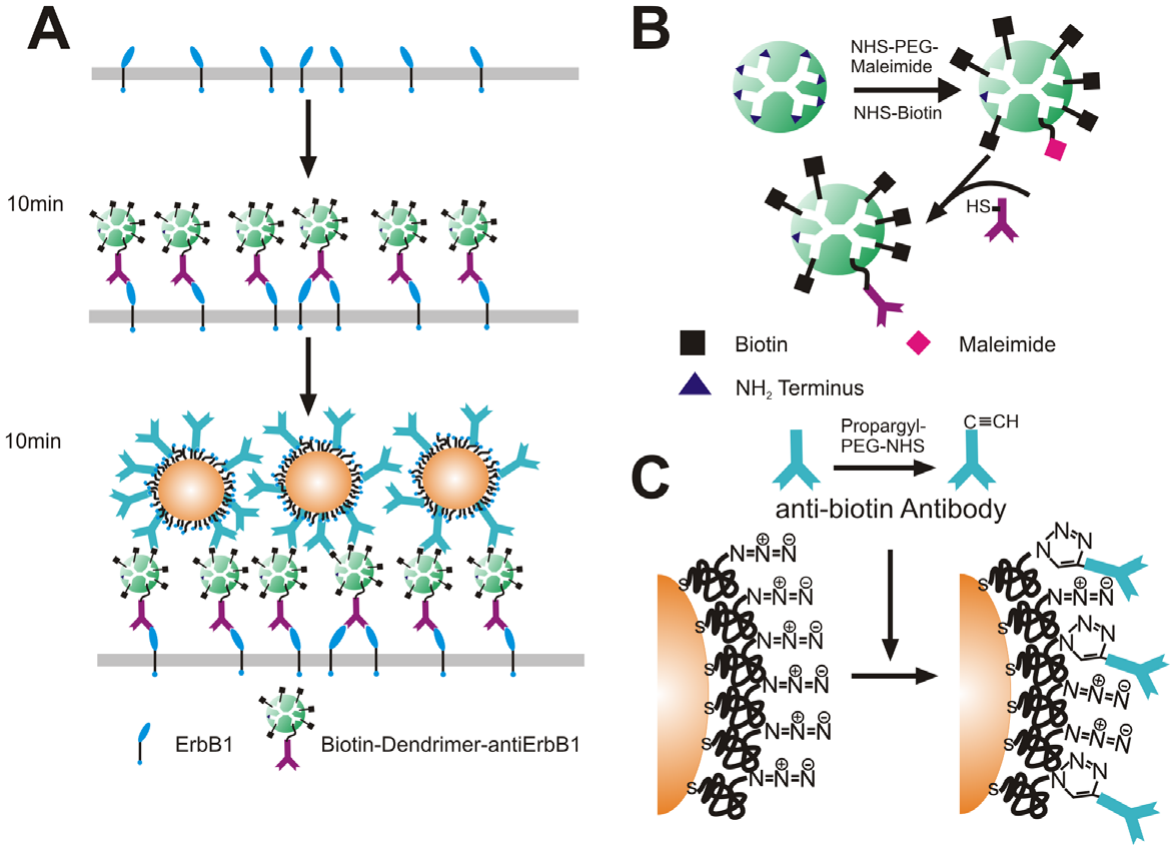
ErbB1 labeling strategy. A. Overview of the dendrimer based labeling strategy. Anti-ErbB1 antibodies tethered to dendrimers functionalized with multiple biotin moieties are targeted to the receptors on the cell surface in a first labeling step. In a second labeling step, anti-biotin antibody functionalized NPs bind to the created binding sites on the cell surface. B. Synthesis of biotin-dendrimer-antibody construct. C. Functionalization of NP surface with anti-biotin antibodies.

In this work we limited the incubation time of the cells with the immunolabels to 10 minutes to avoid a background from endocytosed NPs [58], [59]. To achieve sufficient labeling under these difficult conditions we devised a labeling strategy that incorporates functionalized dendrimers as spacer between the target receptor in the cell surface and the NP label (**Figure 1A**) In this scheme biotinylated dendrimers linked to an anti-ErbB1 antibody are first bound to the receptors on the cell surface to create biotin binding sites. In the second step, Au NPs covalently functionalized with anti-biotin antibodies are then targeted at the created biotin binding sites. One important advantage of this labeling strategy is that the highly branched dendrimers introduce multiple biotins per ErbB1 receptor. This amplification of the number of binding sites is expected to increase the colloidal binding affinity [43].

**Figure 1B** gives details regarding the chemical functionalization of the dendrimers used in this work (also see Methods section). Some of the terminal amines of Poly(amido amine) (PAMAM) dendrimers are partially reacted with N-Hydroxysuccinimide-polyethylene glycol-maleimide (NHS-PEG-maleimide) crosslinkers; the remaining amines are subsequently cross-linked to NHS-biotin. The average maleimide/biotin ratio of the dendrimers used in this work was determined as 1/2 by mass spectrometry. The maleimide group forms stable thioether linkages with the thiol groups in cysteines and facilitates, thus, the crosslinking of the dendrimers with anti-ErbB1 antibodies.

**Figure 1C** illustrates the labeling strategy applied for the Au NPs. We used 60 nm Au NPs as labels in this work as they are bright probes that facilitate high temporal resolutions in optical tracking studies with sufficient contrast. Abulrob *et al.* reported the size of ErbB1 clusters on the cell surface of epithelial cells to lie between 50 and 300 nm with an average diameter of 150±60 nm [9]. Based on these previous observations, we anticipate that 60 nm NPs can efficiently cluster in ErbB1 enriched membrane domains. The NPs labels were functionalized with thiol-polyethylene glycol (PEG)-azide molecules with a molecular weight of 3400 Da. The PEGs enable to covalently bind antibodies to the NP surface and, at the same time, sterically stabilize the NPs against agglomeration in physiological buffers. After pegylation, the NPs are almost charge-neutral (the zeta-potential of the NPs at pH 7.2 is −3 mV), but remain dispersed and do not aggregate in Hanks buffer supplemented with 10 mM HEPES, pH 7.2. The azide groups introduced through the PEGs are subsequently used to covalently bind anti-biotin antibodies modified with propargyl residues *via* a copper catalyzed 1,3-dipolar cycloaddition (see Methods section) [60].

**Figure 2:**
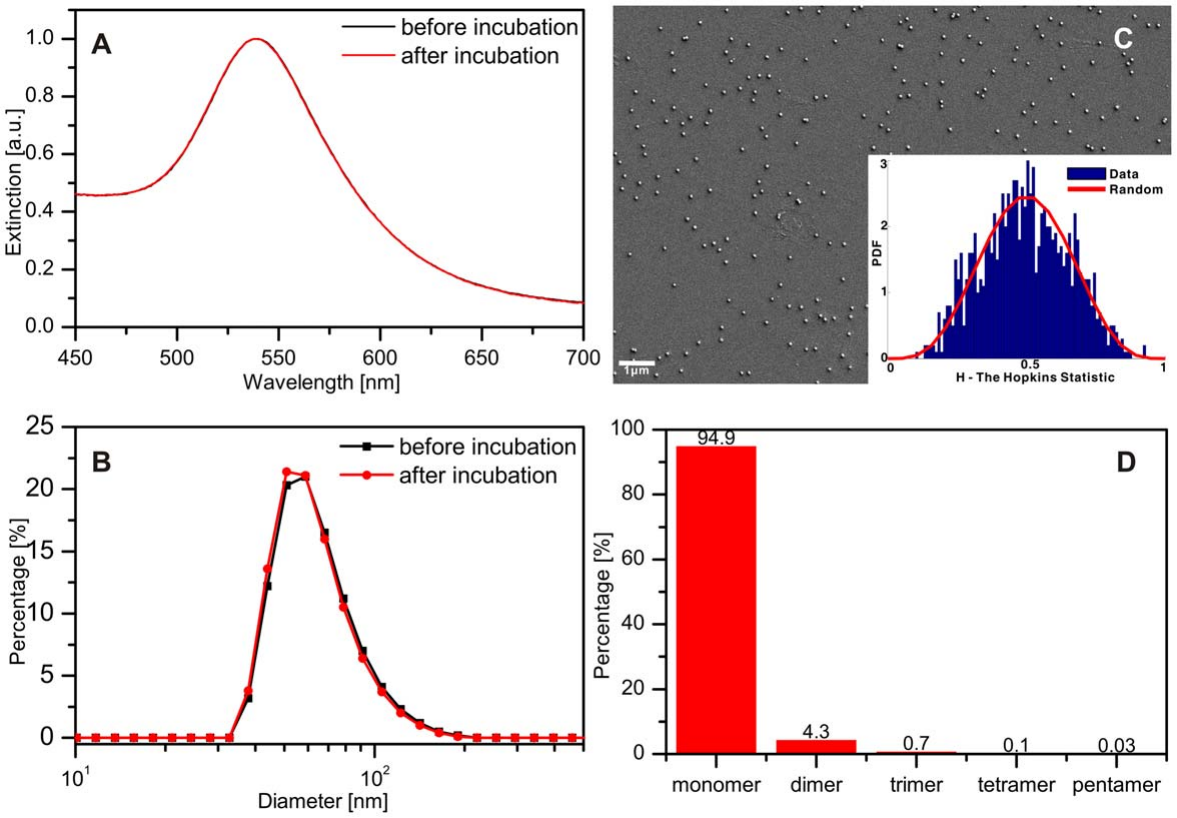
Stability of anti-biotin functionalized NPs. A. UV-Vis spectra of 60 nm NPs functionalized with anti-biotin before and after incubation for 10 min with A431 cells in Hanks buffer with 10 mM HEPES pH 7.2. B. Size distribution as determined by dynamic light scattering. C. SEM image of surface immobilized NPs (on a BSA-biotin functionalized glass substrate) after incubation with cells. The inset shows the Hopkins statistics for the field of view. D. Histogram of the NP association levels after incubation with the cells.

We tested the stability of the resulting anti-biotin antibody functionalized NPs under our experimental conditions. To that end, we incubated the nanoparticles with A431 cells in Hanks buffer supplemented with HEPES for 10 minutes and then recovered the NPs. **Figures 2A** and **B** show the UV-Vis spectra and size distributions determined by dynamic light scattering (DLS) for the NPs before and after incubation. The UV-Vis and DLS data for these two conditions superimpose, confirming that the NPs are stable. This finding is corroborated by an inspection of the NPs recovered after incubation with the cells in the scanning electron microscope (SEM) (**Figure 2C**). We tested for a systematic association of the NPs using the Hopkins test (see Methods). The Hopkins statistics (*H*) can assume values between 0 and 1. A random NP distribution leads to an average *H* value of 0.5, whereas a systematic clustering shifts *H* to higher values. The *H* distribution calculated for the NPs in **Figure 2C** is shown as inset. It reproduces a random distribution (red curve) centered at around 0.5, confirming that the NP are randomly distributed and not clustered. The aggregation statistics for a total of 3476 NPs in **Figure 2D** also shows that the overwhelmingly majority of the NPs are monomeric and that the self-association of the NP immunolabels used in this work is negligible. In the histogram shown in **Figure 2D** dimers and higher oligomers are defined as NP congregations that contain two or more particles with separations below one particle diameter.

### Analysis of the Spatial Distribution of NPs Bound to Cell-Surface ErbB1 in the SEM

**Figure 3:**
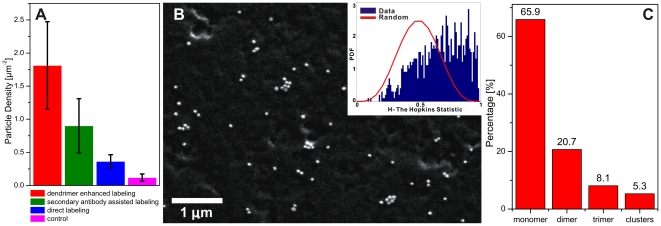
Clustering of NP immunolabels targeted at ErbB1. A. Comparison of immunolabel densities obtained with different labeling strategies: dendrimer enhanced labeling (red), secondary antibody assisted labeling (olive), direct labeling (blue). Controls (dendrimer enhanced labeling in the presence of excess antibodies, see text) are included in magenta. B. Part of an SEM image of a labeled cell surface (dendrimer enhanced strategy). The Hopkins statistics for the full image in the inset shows that the NP distribution is not random but that the NPs show clustering. C. Histogram of the NP cluster sizes on the cell surface.

We compared the NP density on the cellular surface achieved through the dendrimer mediated receptor labeling strategy with that obtained through direct targeting of the receptor with anti-ErbB1 functionalized NPs. Both immunolabels were synthesized using the same ratio of antibodies/NPs, and the cells were incubated with identical concentrations of NPs as determined by the optical density (OD) of the samples (approx. 5×10^10^ particles/mL) for 10 minutes. The cells were subsequently washed with copious amounts of Hanks buffer, fixed, and prepared for inspection in the SEM to determine the average NP cell surface density (see Methods). The histogram in **Figure 3A** shows the NP densities obtained with both labeling approaches. The dendrimer approach achieves a ∼4 times higher NP density on the cellular surface than the alternative “direct” labeling strategy based on anti-ErbB1 functionalized NPs. Control experiments performed with an excess of free competing antibody (anti-biotin, anti-ErbB1, respectively) showed only negligible binding for both labeling strategies, confirming that the observed binding in both cases was ErbB1 specific. We also included the NP density obtained with biotinylated secondary Immunoglobulin G (IgG) antibodies instead of the dendrimers in **Figure 3A**. The average labeling density obtained with the secondary antibody strategy was lower compared to the dendrimer enhanced labeling by a factor of ½.

We attribute the observed enhancement in labeling efficiency observed for the dendrimer strategy to the increased configurational flexibility that results from the additional spacers (dendrimer-antibody construct) and to the amplification of available binding sites on the surface through the creation of multiple biotins. The dendrimer enhanced binding scheme achieves an average NP density of approx. 1.8 NPs/µm^2^ within an incubation time of 10 min. The excerpt from an SEM image of the labeled plasma membrane in **Figure 3B** illustrates that already at these densities the NPs are frequently organized into dimers or higher order oligomers. We confirmed the apparent clustering of the NPs on the cell surface through application of the Hopkins test (see Methods). The distribution of the calculated *H* values of the entire SEM micrograph (334 NPs) for **Figure 3B** (blue) is clearly shifted with regard to the random distribution (red), confirming that the NPs are clustered on the cell surface. We randomly checked ∼20 SEM micrographs of cellular surfaces, all of which indicated a significant clustering of the NP immunolabels.

The stability of the NPs in solution (**Figure 2**) and the fact that the NPs in the clusters in **Figure 3B** are often separated by a visible gap exclude non-specific NP aggregation as cause for the observed NP clustering on the cell surface. Instead, we attribute the observed NP clustering to a heterogeneous ErbB1 topography in the cell surface. The NP clustering is consistent with a preferential enrichment of ErbB1 in signaling domains [61]–[63] that show higher NP binding affinities than surrounding areas. We analyzed the cluster size distribution for a total of 6303 NPs from 8 independent labeling experiments and found that 20.7% of the NPs were organized into dimers, 8.1% into trimers and 5.3% into larger clusters (**Figure 3C**). These data indicate an ErbB1 domain size distribution between ∼60 and ∼250 nm, with an average domain size of ∼110 nm.

While the SEM images in **Figure 3B** provide detailed information about the clustering of the NPs at one specific point of time, they provide no information about the lateral diffusion or structural dynamics of the targeted ErbB1 membrane domains. Sample inspection in the SEM requires a fixation and dehydration of the sample and is not compatible with dynamic tracking studies. PCM, on the other hand, facilitates the detection and approximate sizing of discrete NP clusters in the optical microscope [42]. PCM also enables to track the correlated lateral diffusion of optically colocalized NPs and to simultaneously monitor the configurational dynamics of the NPs within the clusters [36]. Since PCM is an optical microscopy, these studies can be performed with living cells under physiological conditions at 37°C.

### Monitoring the Location, Size, and Structural Dynamics of Individual NP Clusters through PCM

A clustering of NP immunolabels due to co-confinement of multiple NPs in one membrane domain is accompanied by a hybridization of the LSPRs of the individual NPs. The resulting red-shift and broadening of the collective plasmon resonance facilitate a detection of ErbB1 membrane domains through NP clustering in the optical microscope. The scattering spectra, the total scattering cross-sections, and the polarization properties of NP clusters depend sensitively on the exact arrangement and interparticle separation of the electromagnetically coupled NPs [64]–[66]. All of these observables encode valuable information about the confinement of the NPs. The intensity and polarization of the scattered light, in particular, are useful observables since they are experimentally easily accessible even for laterally diffusing clusters [36].

Our experimental strategy to image NP immunolabels targeted at ErbB1 receptors on A431 cells is based on conventional widefield darkfield microscopy [67], [68]. Whitelight is injected into the sample plane at oblique angles using a high numerical aperture (NA = 1.2–1.4) oil darkfield condenser so that only light scattered from the cell surface is collected through the microscope objective. The collected beam is split into two orthogonal polarization channels and imaged on two separate electron multiplying charge coupled devices (EMCCDs) (see Methods) [36]. This approach can track individual diffusing NP clusters on two orthogonal polarization channels, and it simultaneously provides the reduced polarization dichroism (*P*) as function of time and location on the cell surface [69]:
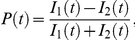
where *I_1_*(*t*) and *I_2_*(*t*) are the integrated scattering intensities from the fitted point-spread-functions for one cluster on the two orthogonal polarization channels at time (*t*). *P* depends on the geometric configuration of a NP cluster and its orientation with regard to the two monitored polarization axes. Together with the total scattering intensity *I_tot_*(*t*) = *I_1_*(*t*)+*I_2_*(*t*), which increases with decreasing separation between coupled nanoparticles, *P* enables to detect configurational changes and rotational motions of NP clusters. Another advantageous characteristic of *P*, in particular, is that even large changes in the refractive index of the ambient medium only lead to relatively moderate changes in *P* [38]. This robustness of *P* against refractive index fluctuations is a plus for plasmon coupling based imaging applications in complex environments.

**Figure 4:**
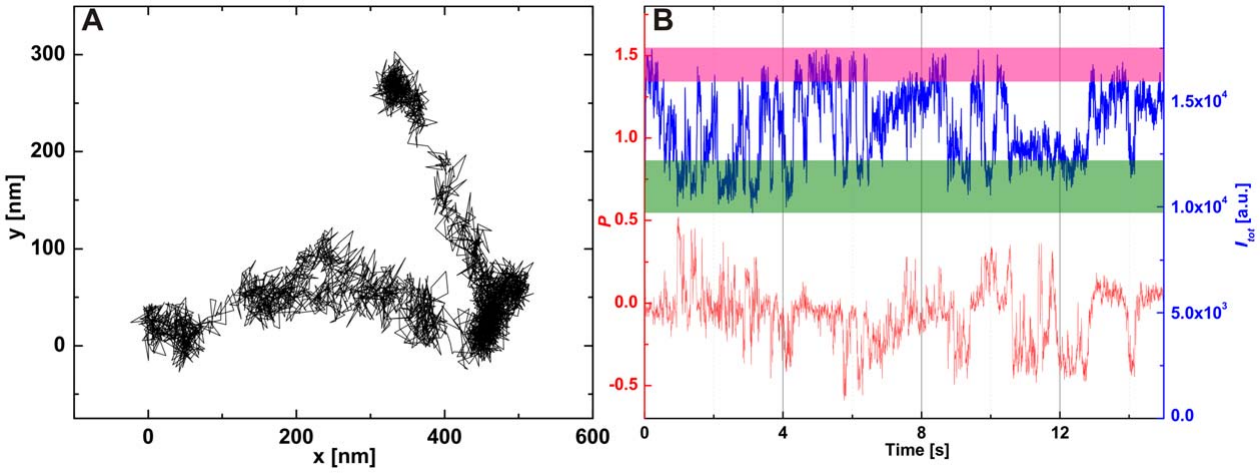
Monitoring the configurational dynamics of laterally diffusing NP clusters through PCM. A. Diffusion trajectory of a NP cluster on the cell surface. B. Reduced polarization dichroism (*P*, red) and total intensity (*I_tot_*, blue) of the light scattered off the diffusing cluster as function of time. We indicated high (pink) and low (olive) intensity levels in the *I_tot_* trajectory. The large fluctuations in *I_tot_* and *P* are characteristic of a rich configurational dynamics in which the NPs of the cluster change their separation and geometric arrangement.

We focused in our experiments on tracking isolated, individual clusters. Despite the low NP labeling density we cannot exclude *a priori* that in some cases non-coupled NPs located in the vicinity of the clusters (within a distance below our experimental resolution) contribute to the detected signal. Non-interacting, spherical NPs provide, however, a constant contribution to *P* and *I*_tot_ and do not interfere with the fluctuations in *P* and *I*_tot_ due to orientational and/or configurational changes of the clusters. A diffusion trajectory and the corresponding *P*(*t*) and *I_tot_*(*t*) values for a representative cluster are shown in **Figure 4A** and **4B**. During our observation time of *t* = 15 s the tracked NP cluster in **Figure 4** does not dissociate. A synchronized diffusion of individual NPs over this extended period of time requires a stabilization of the cluster either by direct attractive interactions between the NPs or by confinement of the NPs to a membrane domain with a high structural integrity, for instance, a membrane “corral” [22]. The total scattering intensity *I_tot_* of the tracked NP cluster (**Figure 4B**) shows fluctuations as function of time indicative of significant changes in the separation between the NPs of the cluster. A continuous reconfiguration of the cluster structure during its diffusion across the cell surface requires some flexibility in the separations of the NPs within the clusters. The observed behavior indicates a hindered diffusion of the NPs within the confined space of a membrane domain that has slightly larger dimensions than the NP cluster. The observed translation of the NP cluster is then the result of an effective lateral diffusion of the confining membrane domain. This interpretation is also consistent with our control experiments (**Figure 2**), which have shown that the NPs are stable and show negligible tendency for self-association.

**Figure 5:**
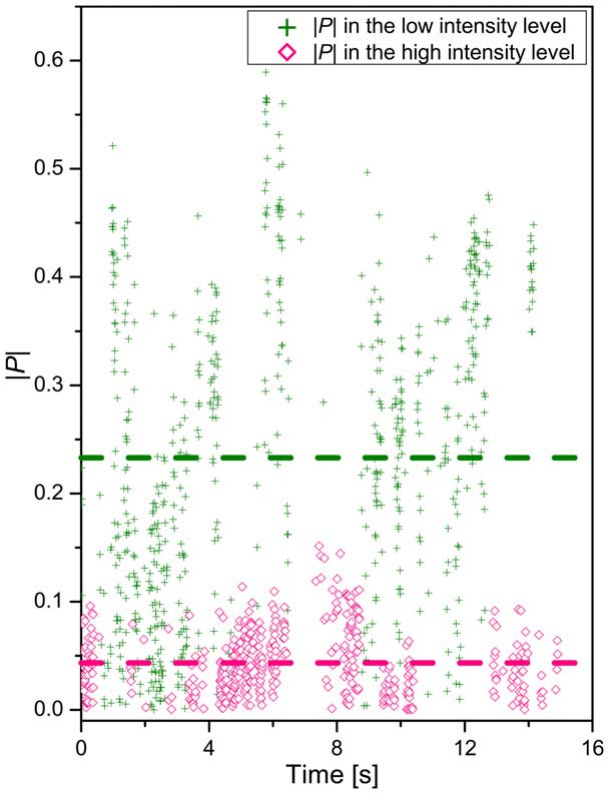
Correlation of |*P*| with high and low intensity (*I_tot_*) configurations. The |*P*| values for the high *I_tot_* configuration for the cluster from **Figure 4** are plotted in pink, the |*P*| values for the low *I_tot_* configuration are plotted in olive.

We marked the high and low total intensity (*I_tot_*) levels in **Figure 4B** pink and olive, respectively. A closer analysis of the correlation of *P* and *I_tot_* reveals that low *I_tot_* values coincide with higher values of the absolute reduced polarization dichroism (|*P*|) than the high *I_tot_* values. In **Figure 5** we plot |*P*| for the high (pink) and low (olive) *I_tot_* levels as function of time. The time-averaged absolute *P* values (|
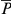
|) for the high (|
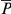
| = 0.044) and low (|
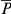
| = 0.233) *I_tot_* values are included as dashed lines. A sharp |
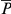
| value distribution close to 0 for the high *I_tot_* level indicates that the light polarization becomes random on our acquisition time scale due to a fast rotational motion (or configurational restructuring) of this cluster. We attribute the remaining low net polarization to a slight polarization of the excitation light in the darkfield optics. In contrast, the |*P*| values in the low *I_tot_* intensity configuration are broadly distributed across the interval |*P|* = [0–0.6] (**Figure 5**), which indicates that the cluster gets transiently trapped in many different configurations and/or orientations on the surface. Together these observations suggest that the NP cluster in the confinement fluctuates between one or several compact configuration(s) with high rotational mobility and bulkier configurations with hindered rotational mobility that remain fixed in space for sufficiently long periods of time to induce a measurable polarizations of the collected light.

**Figure 6:**
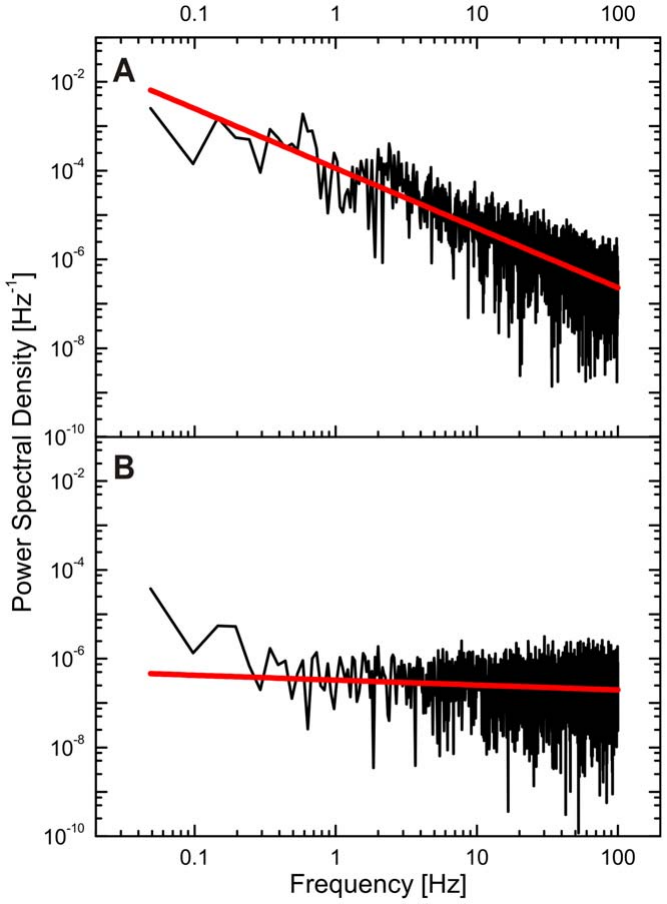
Frequency domain analysis of the reduced polarization dichroism of NP clusters. A. Power spectral density (PSD) of *P* for the NP cluster from **Figure 4**. B. PSD of *P* for a cluster of similar intensity immobilized on a glass substrate.

The configurational dynamics of the clusters can be further quantified through calculation of the power spectral density (PSD) of the *P* trajectory. Any displacement of the NPs within one cluster relative to each other leads to time-dependent fluctuations in the interparticle separations as well as geometric configuration and, thus, contributes to the “noise” in the *P* trajectory. We calculated the PSD of the *P* trajectory shown in **Figure 4B**. The PSD (**Figure 6A**) falls off as 1/f^1.3^, which is slower than expected for Brownian noise (1/f^2^), and is, therefore, consistent with a constrained diffusion within a laterally diffusing domain. For comparison we show the PSD of *P* for a single immobilized NP cluster in **Figure 6B**. Due to the absence of any configurational dynamics, the PSD of an individual, immobilized NP cluster is dominated by electronic noise. Consequently, the PSD is flat across the monitored frequency range as expected for white noise.

Both the time-domain and frequency-domain PCM data indicate that the NPs are electromagnetically coupled due to the confinement of multiple NPs to a membrane area that is of similar size as the total integrated physical cross-section of the NP cluster. The latter can be approximated through comparison of the average scattering intensity of the NP cluster with that of individual 60 nm Au NPs. For the cluster in **Figure 4** we find that the cluster comprises 2–3 individual NPs, and we conclude that the membrane domain that accommodates the NP cluster has an approximate diameter between 120–180 nm. The comparison of the cluster intensity with that of individual NPs somewhat overestimates the size of the clusters since it does not take into account the increase in scattering intensity due to plasmon coupling in the cluster. These effects can be accounted for in a more complex data analysis [42], but for most practical applications an approximate sizing based on the scattering intensity will be sufficient.

**Figure 7:**
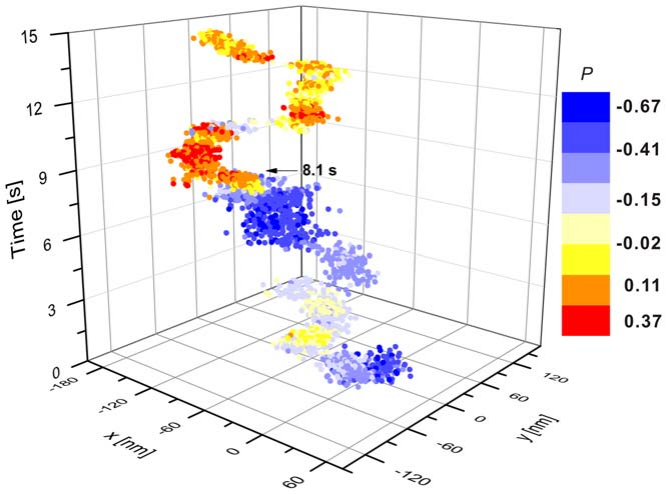
Mapping the spatiotemporal history of NP cluster configurations through PCM. *P* as function of time and location during the lateral translation of a NP cluster comprising three to four 60 nm diameter Au NPs. At t = 8.1 s the P value shows a systematic shift indicative of a change in the cluster configuration.

Although the diffusion of the NPs comprising the cluster shown in **Figure 4** are clearly hindered, the seemingly randomly occurring large amplitude *I_tot_* and *P* fluctuations are evidence of some residual mobility of the NPs within the confining domain. Other clusters showed a significant lower degree of structural flexibility. This is exemplified in **Figure 7** where we plot the calculated *P* values of another NP cluster as function of location and time. Based on the average scattering intensity, we estimate that this cluster comprises 3–4 NPs, corresponding to an approximate size of the confinement between 180–240 nm. The *P* values of this cluster are predominantly negative for the first part of the trajectory but at *t*≈8.1 s the *P* values abruptly shift to positive values and remain positive for most of the remaining observation time. This behavior indicates a confined NP cluster with strongly constricted structural flexibility for *t*<8.1 s and *t*>8.1 s.

The systematic shift in *P* at *t*≈8.1 s marks the transition from one stable into a second stable NP cluster configuration and could be the result of a morphological change of the underlying membrane structure which patterns the NP clusters. A slow lateral domain diffusion, as is evident from the translation of the NP clusters in **Figures 4A** & **7**, has been associated with the dynamic restructuring of the membrane supporting cytoskeleton before [18]. To investigate the effective diffusion of entire membrane domains in more detail, we will in the next section quantitate the correlated diffusion of entire NP clusters.

### Analysis of the Lateral Diffusion of NP Labeled Membrane Domains

**Figure 8:**
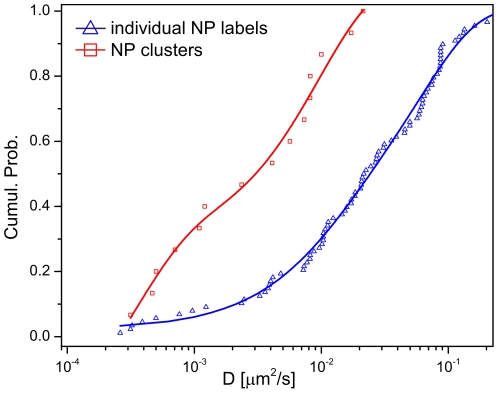
Comparison of diffusion coefficient (*D*) distributions for individual NPs and NP clusters. The graphs show the cumulative distributions of the *D* values of individual NP labels (*blue*) and of NP clusters (*red*). The *D* value distribution of the clusters is systematically shifted to lower values when compared with that of individual NPs.

PCM provides all the information available from conventional NP tracking and lends itself naturally to quantifying the lateral diffusion of the NP clusters. The experimental mean diffusion coefficient (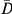) for individual NPs was determined as 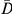 = |0.048±0.065| µm^2^/s (measured at a temporal resolution of 200 Hz). This 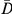 value is in good agreement with previous ErbB1 tracking studies [16], [28], [70]–[72], and it does not significantly decrease if it is evaluated at lower frame rates (e.g., 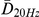 = |0.040±0.066| µm^2^/s). The distribution of the diffusion coefficient (*D*) values for individual NPs in our study is, however, very broad (**Figure 8**). *A priori*, we cannot exclude that the tail of the distribution at low *D* values results from receptor crosslinking through multivalent NPs. Although the obtained 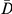 value for individual NPs might underestimate the dynamics of individual receptors, a comparison of the NP and NP cluster *D* value distributions, which are both included in **Figure 8**, unambiguously shows that the diffusion coefficient distribution of NP clusters is systematically shifted to lower *D* values. This analysis confirms that NP clusters diffuse significantly slower than individual NPs. We obtained a 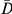 value for 15 tracked clusters of 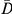 = |0.0054±0.0064| µm^2^/s.

Consistent with this overall shift in diffusion coefficients, both of the clusters shown in **Figures 4** and **7** exhibit significantly slower diffusion coefficients than the individual NP labeled ErbB1 receptors. The cluster shown in **Figure 4**, which comprises 2–3 NPs and the cluster in **Figure 7** with 3–4 NPs show almost identical *D* values of 7×10^−4^ µm^2^/s. We ascribed the formation of NP clusters at overall low NP labeling densities to the existence of high affinity binding sites in ErbB1 enriched membrane domains. The observed slow lateral diffusion of these membrane domains is consistent with previous studies by Andrews *et al.* [18], in which the authors showed that a dynamic reorganization of the cytoskeleton network on the time scale of seconds to tens of seconds leads to an effective lateral diffusion of the enclosed membrane meshwork. While the work by Andrews *et al.* focused on the diffusion of the high affinity IgE receptor (FcεRI) in micron sized membrane compartments defined by the actin network of the cytoskeleton, the ErbB1 enriched domains detected by NP clustering in this work are sub-micron. With typical diameters between 0.1–0.3 µm the detected stable ErbB1 domains are more similar in size to the corrals in the meshwork formed by non-actin based components of the cytoskeleton, such as spectrin [23], [73]. Our PCM studies indicate a continuous restructuring of the tracked ErbB1 enriched membrane domains on the second to tens of seconds time scale, resulting in an ErbB1 distribution on the cell surface that is heterogeneous in both space and time.

### Conclusions

We have applied a dendrimer amplified binding strategy to label unliganded ErbB1 receptors on the surface of living A431 cells. Inspection of these samples in the SEM revealed that already under relatively low labeling levels (nanoparticle density ≈ 1.8 NPs/µm^2^), the NPs are substantially associated into oligomers on the cell surface. We found that the NP cluster sizes range from ∼60 to ∼250 nm with an average domain size of ∼110 nm. The observation of NP clustering, together with the fact that under identical experimental conditions no agglomeration of the NPs in solution was observed, confirms that the NP clustering is the result of a heterogeneous distribution of ErbB1 density on the cell surface. Au NPs are multimodal probes that can be imaged in the optical microscope. We applied polarization resolved plasmon coupling microscopy (PCM) to detect NP clusters and to characterize the structural dynamics of the NPs in their membrane confinement with a frame rate of 200 frames/s. The obtained information about the relative mobility of the NPs within their confinements and the total scattering intensity of the co-localized NPs facilitated an approximate sizing of individual membrane domains. We tracked individual NP cluster containing domains and found that the ErbB1 enriched domains show a lateral diffusion (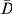 = |0.0054±0.0064| µm^2^/s), which is nearly one order of magnitude slower than that of individual NP labeled ErbB1 receptors (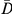 = |0.048±0.065| µm^2^/s). The local enrichment of ErbB1 in sub-micron confinements and the slow effective diffusion of these domains are consistent with a patterning of the ErbB1 density on the tens of nanometer length scale by continuously restructuring plasma membrane domains. The spatial distribution of the ErbB1 density (and of other transmembrane receptors) plays a potentially important role in coordinating and controlling cell signaling. We have demonstrated that PCM enables to visualize ErbB1 clustering in native plasma membranes of living cells and that it provides insight into the lateral dynamics of individual ErbB1 membrane domains.

## Materials and Methods

### Materials

We used the following materials without purification: 60 nm Au colloids (Ted Pella); thiol-polyethylene glycol-azide (N_3_-(CH_2_CH_2_O)_77_-CH_2_CH_2_–SH, MW: 3400 Da) (NANOCS Inc); propargyl dPEG-NHS ester (Quanta Biodesign); monoclonal anti-epidermal growth factor receptor antibody (199.12) (Lab Vision); anti-biotin affinity isolated antigen specific antibody (Sigma); PAMAM dendrimer, ethylenediamine core, generation 1.0 (Aldrich); biotin N-hydroxysuccinimide ester (biotin-NHS) (Sigma); NHS-PEG_6_-Maleimide (Thermo Scientific); l-ascorbic acid (Aldrich); copper(II) sulfate pentahydrate (Aldrich); triethylamine (Sigma-Aldrich); We used Zeba™ spin desalting columns (7 K MWCO) from Thermo Scientific.

### Synthesis and Characterization of Dendrimer Construct

PAMAM G-1 dendrimers were dissolved in DMF at a concentration of 7 mM, and triethylamine (0.01 mM) and NHS-(EG)_6_-Maleimide (10 mM) were added to the dendrimer with mixing. After 2 h incubation at room temperature, biotin-NHS (50 mM) were then added to the reaction and incubated for 2 h. Tris buffer was added to quench the reaction. 1 µL of this maleimide-dendrimer-biotin construct was then incubated overnight at 4°C with 500 µL 200 nM Anti-ErbB1 in the imaging buffer (Hanks balanced salt solution (HBSS) and 10 mM HEPES pH 7.2). Then the excess maleimide-dendrimer-biotin was removed using a size-exclusion column (MWCO: 7 K).

The obtained biotin-dendrimer-maleimide construct was diluted in 400 µL DI water for characterization by mass spectrometry on a Waters Qtof (hybrid quadrupolar/time-of-flight) API US system by electrospray (ESI) in the positive mode. ESI-MS: 1114.93 (biotin_2_-dendrimer-maleimide_3_^3+^, calcd 1114.99); 1125.26 (biotin_4_-dendrimer-maleimide_2_^2+^, calcd 1125.66); 836.22 (biotin_6_-dendrimer-maleimide^4+^, calcd 836.02).

### Nanoparticle Functionalization

The anti-ErbB1 and anti-biotin conjugated Au NPs were functionalized as follows: 5 µL thiol-PEG-azide (10 mM) were incubated with 60 nm Au NPs (2.6×10^10^ particles/mL) overnight at ambient temperature. The PEGylated Au NPs were then purified though repeated centrifugation (2500 rpm, 3×) and re-suspension in DI water (18.2 MΩ). The final volume of the NP solution was 20 µL. 2 µL of propargyl-PEG-NHS ester solution (100 mg/mL in DMSO) was added to 100 µL 1 mg/mL biotin antibody or ErbB1 antibody PBS solution (pH 7.2), respectively. The reaction was carried out in an ice bath for 6 h. Then the excess propargyl-PEG-NHS was removed using a size-exclusion column (MWCO: 7 K).

200 µL of 0.25 mg/mL functionalized antibody were then incubated overnight at 4°C with PEGylated Au NPs. This reaction was catalyzed by 20 nmol copper (II) sulfate and 100 nmol ascorbic acid. The final antibody-Au NP conjugates were washed three times. The cleaned immunolabels were re-suspended in the imaging buffer to a final concentration of 5×10^10^ particles/mL.

### Cell Culturing

A431 cells (ATCC) were cultured in the advanced Dulbecco's Modified Eagle Medium supplemented with 10% fetal bovine serum, 50 units/ml penicillin, 50 µg/ml streptomycin and 2 mM L-glutamine at 37°C in a humidified, 5% CO_2_ atmosphere. For immunolabeling and darkfield imaging the cells were grown on glass coverslips to approximately 40% confluency. The cells used for SEM imaging were seeded and grown on 1×1 cm silicon chips under the same culturing conditions.

### Immunolabeling and Darkfield Microscopy

In the dendrimer mediated labeling strategy the antiErbB1-dendrimer-biotin constructs were incubated with A431 cells in a home-made glass flow-chamber at 37°C for 10 minutes. After rinsing the chamber with imaging buffer, the anti-biotin antibody functionalized NPs were incubated with cells for additional 10 minutes at 37°C. All live cell imaging experiments were carried on an inverted darkfield microscope (Olympus IX71) equipped with a cage incubator. The sample was illuminated by Xenon white light through a NA 1.2–1.4 oil darkfield condenser. The scattered light was collected by an oil-immersed 60× (numerical aperture, NA = 0.65) objective, magnified by an additional factor of 1.6× and split into two orthogonal light channels through a polarizing beam splitter. The two beams with orthogonal polarization were reimaged on two electron multiplying charge coupled devices (EMCCDs). We used Andor IxonEM^+^ detectors with a maximum detection area of 128×128 pixels and a pixel size of 30 µm×30 µm. All tracking experiments were performed with frame rates of 200 frames/s.

### Particle Tracking and Trajectory Analysis

The locations of the NPs and NP clusters on the two orthogonal polarization channels were obtained by fitting their point-spread-functions (PSFs) to two-dimensional Gaussians. At frame rates of 200 Hz, the location precision was σ≈33 nm on both channels. The individual scatterers were independently tracked on the two polarization channels and the integrated intensities of their PSFs were used to calculate the reduced polarization dichroism (*P*) in each frame. The diffusion coefficients (*D*) of individual scatterers were calculated from the trajectories recorded on one of the polarization channels.

The *D* values of individual scatterers were determined by fitting the mean square displacement (MSD) versus time lag (*t*) relationship by a linear fit of the form MSD(*t*) = 4*Dt*+*β*, where the optimal number of MSD points used was determined as a function of localization accuracy, diffusion coefficient and other experimental parameters, as previously described [74]. The values and errors of mean diffusion coefficients (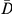) are given as “mean ± standard deviation” throughout the text.

### Nanoparticle Surface Density Calculations

Particle number and locations were determined by home-written Matlab codes that find local maxima in the individual SEM images. Particle surface densities were then averaged over membrane areas of approximately 20×14 µm^2^.

### Spatial Clustering Analysis Using Hopkins Statistics

We chose the Hopkins Statistics as a quantitative measure to test the Complete Spatial Randomness (CSR) hypothesis by comparing the nearest neighbor distance distribution of *m* random sampling points (*U*) and *m* random selected particles (*W*) [75]. The Hopkins statistics (*H*) is defined as:
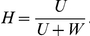
The probability density function for *H* of *m* random sampling points under the CSR follows the beta distribution:
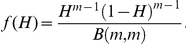
The particles are randomly distributed when *H* value distribution peaks around 0.5 and are clustered when *H* value distribution skews to 1.

## References

[1] Warren CM, Landgraf R (2006). Signaling through ErbB receptors: Multiple layers of diversity and control.. Cellular Signalling.

[2] Yarden Y, Sliwkowski MX (2001). Untangling the ErbB signalling network.. Nature Reviews Molecular Cell Biology.

[3] Ozcan F, Klein P, Lemmon MA, Lax I, Schlessinger J (2006). On the nature of low- and high-affinity EGF receptors on living cells.. Proceedings of the National Academy of Sciences of the United States of America.

[4] Schlessinger J (2002). Ligand-induced, receptor-mediated dimerization and activation of EGF receptor.. Cell.

[5] Clayton AHA, Orchard SG, Nice EC, Posner RG, Burgess AW (2008). Predominance of activated EGFR higher-order oligomers on the cell surface.. Growth Factors.

[6] Clayton AHA, Tavarnesi ML, Johns TG (2007). Unligated epidermal growth factor receptor forms higher order oligomers within microclusters on A431 cells that are sensitive to tyrosine kinase inhibitor binding.. Biochemistry.

[7] Clayton AHA, Walker F, Orchard SG, Henderson C, Fuchs D (2005). Ligand-induced dimer-tetramer transition during the activation of the cell surface epidermal growth factor receptor-a multidimensional microscopy analysis.. Journal of Biological Chemistry.

[8] Szabo A, Horvath G, Szollosi J, Nagy P (2008). Quantitative characterization of the large-scale association of ErbB1 and ErbB2 by flow cytometric homo-FRET measurements.. Biophysical Journal.

[9] Abulrob A, Lu Z, Baumann E, Vobornik D, Taylor R (2010). Nanoscale imaging of epidermal growth factor receptor clustering effects of inhibitors.. Journal of Biological Chemistry.

[10] Nagy P, Jenei A, Kirsch AK, Szollosi J, Damjanovich S (1999). Activation-dependent clustering of the ErbB2 receptor tyrosine kinase detected by scanning near-field optical microscopy.. Journal of Cell Science.

[11] Yang S, Raymond-Stintz MA, Ying W, Zhang J, Lidke DS (2007). Mapping ErbB receptors on breast cancer cell membranes during signal transduction.. Journal of Cell Science.

[12] Ariotti N, Liang H, Xu YF, Zhang YQ, Yonekubo Y (2010). Epidermal growth factor receptor activation remodels the plasma membrane lipid environment to induce nanocluster formation.. Molecular and Cellular Biology.

[13] Bader AN, Hofman EG, Voortman J, Van Bergen En Henegouwen PMP, Gerritsen HC (2009). Homo-FRET imaging enables quantification of protein cluster sizes with subcellular resolution.. Biophysical Journal.

[14] Balzani V, Ceroni P, Gestermann S, Kauffmann C, Gorka M (2000). Dendrimers as fluorescent sensors with signal amplification.. Chemical Communications.

[15] Hsieh MY, Yang S, Raymond-Stinz MA, Steinberg S, Vlachos DG (2008). Stochastic simulations of ErbB homo and heterodimerisation: Potential impacts of receptor conformational state and spatial segregation.. Iet Systems Biology.

[16] Low-Nam ST, Lidke KA, Cutler PJ, Roovers RC, Van Bergen En Henegouwen PMP (2011). ErbB1 dimerization is promoted by domain co-confinement and stabilized by ligand binding.. Nature Structural & Molecular Biology.

[17] Mayawala K, Vlachos DG, Edwards JS (2005). Heterogeneities in EGF receptor density at the cell surface can lead to concave up scatchard plot of EGF binding.. FEBS Letters.

[18] Andrews NL, Lidke KA, Pfeiffer JR, Burns AR, Wilson BS (2008). Actin restricts FcεRI diffusion and facilitates antigen-induced receptor immobilization.. Nature Cell Biology.

[19] Kusumi A, Nakada C, Ritchie K, Murase K, Suzuki K (2005). Paradigm shift of the plasma membrane concept from the two-dimensional continuum fluid to the partitioned fluid: High-speed single-molecule tracking of membrane molecules.. Annual Review of Biophysics and Biomolecular Structure.

[20] Lillemeier BF, Pfeiffer JR, Surviladze Z, Wilson BS, Davis MM (2006). Plasma membrane-associated proteins are clustered into islands attached to the cytoskeleton.. Proceedings of the National Academy of Sciences of the United States of America.

[21] Saxton MJ (1989). The spectrin network as a barrier to lateral diffusion in erythrocytes - a percolation analysis.. Biophysical Journal.

[22] Sheetz MP (1983). Membrane skeletal dynamics - role in modulation of red-cell deformability, mobility of transmembrane proteins, and shape.. Seminars in Hematology.

[23] Tang Q, Edidin M (2003). Lowering the barriers to random walks on the cell surface.. Biophysical Journal.

[24] Tsuji A, Ohnishi S (1986). Restriction of the lateral motion of band-3 in the erythrocyte-membrane by the cytoskeletal network - dependence on spectrin association state.. Biochemistry.

[25] Chen Y, Yang B, Jacobson K (2004). Transient confinement zones: A type of lipid raft?. Lipids.

[26] Marmor MD, Skaria KB, Yarden Y (2004). Signal transduction and oncogenesis by ErbB/HER receptors.. International Journal of Radiation Oncology Biology Physics.

[27] Nagy P, Vereb G, Sebestyen Z, Horvath G, Lockett SJ (2002). Lipid rafts and the local density of ErbB proteins influence the biological role of homo- and heteroassociations of ErbB2.. Journal of Cell Science.

[28] Chung I, Akita R, Vandlen R, Toomre D, Schlessinger J (2010). Spatial control of EGF receptor activation by reversible dimerization on living cells.. Nature.

[29] Douglass AD, Vale RD (2005). Single-molecule microscopy reveals plasma membrane microdomains created by protein-protein networks that exclude or trap signaling molecules in t cells.. Cell.

[30] Nagy P, Claus J, Jovin TM, Arndt-Jovin DJ (2010). Distribution of resting and ligand-bound ErbB1 and ErbB2 receptor tyrosine kinases in living cells using number and brightness analysis.. Proceedings of the National Academy of Sciences of the United States of America.

[31] Ha T, Enderle T, Ogletree DF, Chemla DS, Selvin PR (1996). Probing the interaction between two single molecules: Fluorescence resonance energy transfer between a single donor and a single acceptor.. Proceedings of the National Academy of Sciences of the United States of America.

[32] Lakowicz JR (1999). Principles of fluorescence spectroscopy.

[33] de Lange F, Cambi A, Huijbens R, de Bakker B, Rensen W (2001). Cell biology beyond the diffraction limit: Near-field scanning optical microscopy.. Journal of Cell Science.

[34] Hell SW (2007). Far-field optical nanoscopy.. Science.

[35] Subach FV, Patterson GH, Manley S, Gillette JM, Lippincott-Schwartz J (2009). Photoactivatable mCherry for high-resolution two-color fluorescence microscopy.. Nature Methods.

[36] Rong GX, Wang HY, Reinhard BM (2010). Insights from a nanoparticle minuet: Two-dimensional membrane profiling through silver plasmon ruler tracking.. Nano Letters.

[37] Rong GX, Wang HY, Skewis LR, Reinhard BM (2008). Resolving sub-diffraction limit encounters in nanoparticle tracking using live cell plasmon coupling microscopy.. Nano Letters.

[38] Wang HY, Reinhard BM (2009). Monitoring simultaneous distance and orientation changes in discrete dimers of DNA linked gold nanoparticles.. Journal of Physical Chemistry C.

[39] Yang LL, Wang HY, Yan B, Reinhard BM (2010). Calibration of silver plasmon rulers in the 1–25 nm separation range: Experimental indications of distinct plasmon coupling regimes.. Journal of Physical Chemistry C.

[40] Jain PK, El-Sayed MA (2007). Universal scaling of plasmon coupling in metal nanostructures: Extension from particle pairs to nanoshells.. Nano Letters.

[41] Su KH, Wei QH, Zhang X, Mock JJ, Smith DR (2003). Interparticle coupling effects on plasmon resonances of nanogold particles.. Nano Letters.

[42] Wang HY, Rong GX, Yan B, Yang LL, Reinhard BM (2011). Optical sizing of immunolabel clusters through multispectral plasmon coupling microscopy.. Nano Letters.

[43] Wang J, Boriskina SV, Wang HY, Reinhard BM (2011). Illuminating epidermal growth factor receptor densities on filopodia through plasmon coupling.. ACS Nano.

[44] Aaron J, Travis K, Harrison N, Sokolov K (2009). Dynamic imaging of molecular assemblies in live cells based on nanoparticle plasmon resonance coupling.. Nano Letters.

[45] Crow MJ, Grant G, Provenzale JM, Wax A (2009). Molecular imaging and quantitative measurement of epidermal growth factor receptor expression in live cancer cells using immunolabeled gold nanoparticles.. American Journal of Roentgenology.

[46] Crow MJ, Seekell K, Ostrander JH, Wax A (2011). Monitoring of receptor dimerization using plasmonic coupling of gold nanoparticles.. ACS Nano.

[47] Yguerabide J, Yguerabide EE (1998). Light-scattering submicroscopic particles as highly fluorescent analogs and their use as tracer labels in clinical and biological applications - ii. Experimental characterization.. Analytical Biochemistry.

[48] Yguerabide J, Yguerabide EE (1998). Light-scattering submicroscopic particles as highly fluorescent analogs and their use as tracer labels in clinical and biological applications - i. Theory.. Analytical Biochemistry.

[49] Brinkers S, Dietrich HRC, Stallinga S, Mes JJ, Young IT (2010). Single molecule detection of tuberculosis nucleic acid using dark field tethered particle motion..

[50] Sowa Y, Steel BC, Berry RM (2010). A simple backscattering microscope for fast tracking of biological molecules.. Review of Scientific Instruments.

[51] Ueno H, Nishikawa S, Iino R, Tabata KV, Sakakihara S (2010). Simple dark-field microscopy with nanometer spatial precision and microsecond temporal resolution.. Biophysical Journal.

[52] Yang Y-H, Nam J-M (2009). Single nanoparticle tracking-based detection of membrane receptor-ligand interactions.. Analytical Chemistry.

[53] Murase K, Fujiwara T, Umemura Y, Suzuki K, Iino R (2004). Ultrafine membrane compartments for molecular diffusion as revealed by single molecule techniques.. Biophysical Journal.

[54] Suzuki K, Ritchie K, Kajikawa E, Fujiwara T, Kusumi A (2005). Rapid hop diffusion of a G-protein-coupled receptor in the plasma membrane as revealed by single-molecule techniques.. Biophysical Journal.

[55] Sheets ED, Lee GM, Simson R, Jacobson K (1997). Transient confinement of a glycosylphosphatidylinositol-anchored protein in the plasma membrane.. Biochemistry.

[56] VanTeeffelen JW, Brands J, Stroes ES, Vink H (2007). Endothelial glycocalyx: Sweet shield of blood vessels.. Trends in Cardiovascular Medicine.

[57] Prescher JA, Dube DH, Bertozzi CR (2004). Chemical remodelling of cell surfaces in living animals.. Nature.

[58] Carpenter G (1987). Receptors for epidermal growth-factor and other polypeptide mitogens.. Annual Review of Biochemistry.

[59] Wiley HS, Shvartsman SY, Lauffenburger DA (2003). Computational modeling of the EGF-receptor system: A paradigm for systems biology.. Trends in Cell Biology.

[60] Kolb HC, Finn MG, Sharpless KB (2001). Click chemistry: Diverse chemical function from a few good reactions.. Angewandte Chemie-International Edition.

[61] Mineo C, Gill GN, Anderson GW (1999). Regulated migration of epidermal growth factor receptor from caveolae.. Journal of Biological Chemistry.

[62] Roepstorff K, Thomsen P, Sandvig K, van Deurs B (2002). Sequestration of epidermal growth factor receptors in non-caveolar lipid rafts inhibits ligand binding.. Journal of Biological Chemistry.

[63] Saffarian S, Li Y, Elson EL, Pike LJ (2007). Oligomerization of the EGF receptor investigated by live cell fluorescence intensity distribution analysis.. Biophysical Journal.

[64] Reinhard BM, Siu M, Agarwal H, Alivisatos AP, Liphardt J (2005). Calibration of dynamic molecular rule based on plasmon coupling between gold nanoparticles.. Nano Letters.

[65] Yan B, Boriskina SV, Reinhard BM (2011). Optimizing gold nanoparticle cluster configurations (n< = 7) for array applications.. Journal of Physical Chemistry C.

[66] Yan B, Boriskina SV, Reinhard BM (2011). Design and implementation of noble metal nanoparticle cluster arrays for plasmon enhanced biosensing.. Journal of Physical Chemistry C.

[67] Schultz S, Smith DR, Mock JJ, Schultz DA (2000). Single-target molecule detection with nonbleaching multicolor optical immunolabels.. Proceedings of the National Academy of Sciences of the United States of America.

[68] Sonnichsen C, Franzl T, Wilk T, von Plessen G, Feldmann J (2002). Drastic reduction of plasmon damping in gold nanorods.. Physical Review Letters.

[69] Wei CYJ, Lu CY, Kim YH, Bout DAV (2007). Determining if a system is heterogeneous: The analysis of single molecule rotational correlation functions and their limitations.. Journal of Fluorescence.

[70] Kusumi A, Sako Y, Yamamoto M (1993). Confined lateral diffusion of membrane-receptors as studied by single-particle tracking (nanovid microscopy) - effects of calcium-induced differentiation in cultured epithelial-cells.. Biophysical Journal.

[71] Orr G, Hu DH, Ozcelik S, Opresko LK, Wiley HS (2005). Cholesterol dictates the freedom of EGF receptors and HER2 in the plane of the membrane.. Biophysical Journal.

[72] Xiao ZY, Zhang W, Yang Y, Xu L, Fang XH (2008). Single-molecule diffusion study of activated EGFR implicates its endocytic pathway.. Biochemical and Biophysical Research Communications.

[73] Takeuchi M, Miyamoto H, Sako Y, Komizu H, Kusumi A (1998). Structure of the erythrocyte membrane skeleton as observed by atomic force microscopy.. Biophysical Journal.

[74] Michalet X (2010). Mean square displacement analysis of single-particle trajectories with localization error: Brownian motion in an isotropic medium.. Physical Review E.

[75] Jain AK, Dubes RC (1988). Algorithms for clustering data.

